# Retraction Note: Long non-coding RNA TPTEP1 inhibits hepatocellular carcinoma progression by suppressing STAT3 phosphorylation

**DOI:** 10.1186/s13046-023-02700-6

**Published:** 2023-05-16

**Authors:** Hongda Ding, Junpeng Liu, Ruoyao Zou, Pengrui Cheng, Yang Su

**Affiliations:** Department of the fifth General Surgery, ShengJing Hospital of China Medical University, No. 36 Sanhao road, Shenyang, 110004 China

**Retraction Note:** ***J Exp Clin Cancer Res*** **38, 189 (2019)**

10.1186/s13046-019-1193-0

The Editor-in-Chief has retracted this article. After publication, concerns were raised regarding the data presented in the figures. Specifically:

- In Fig. 3e, the right image appears highly similar to Fig. 3e left in [1].
- In Fig. 3c, plot 1 appears highly similar to plot 2, and plot 3 appears highly similar to plot 6.
- LV-control in Fig. 5d appears highly similar to shRNA control in Fig. S2b.

Additionally, the authors used SMMC-7721, QGY-7703 and L02 cell lines, which have been reported to be contaminated with HeLa cells.

The authors have stated that the data duplication was caused by simple errors, and the overlap with [1] occurred due to shared resources. However, they have been unable to provide sufficient raw data from the original experiments to fully address these concerns.

Due to the high number of image concerns and the use of unsuitable cell models for hepatocellular carcinoma, the Editor-in-Chief no longer has confidence in the presented data.

Hongda Ding agrees to this retraction. Junpeng Liu, Ruoyao Zou, Pengrui Cheng and Yang Su have not responded to any correspondence from the editor or publisher about this retraction.
